# Estimating a constant WTP for a QALY—a mission impossible?

**DOI:** 10.1007/s10198-017-0929-z

**Published:** 2017-09-21

**Authors:** Björn Sund, Mikael Svensson

**Affiliations:** 1 Swedish Civil Contingencies Agency (MSB), 651 81 Karlstad, Sweden; 2 Department of Economics, Karlstad University, Karlstad, Sweden; 3 Health Metrics Unit, Sahlgrenska Academy, University of Gothenburg, Gothenburg, Sweden

**Keywords:** Willingness to pay, Quality-adjusted life year, Contingent valuation, Scope sensitivity, D61, H43, I31

## Abstract

Economic evaluations are an important input to decision-making and priority-setting in the health care sector. Measuring preferences for health improvements, as the demand-side value (willingness to pay) of gaining a quality-adjusted life year (QALY), is one relevant component in the interpretation of the results from health economic evaluations. Our article addresses whether willingness to pay for a QALY (WTP-Q) is sensitive to the size of the health differences and the probability for improvement. We use data from a contingent valuation survey based on 1400 respondents conducted in the spring of 2014. The results show that the expectation of sensitivity to scope, or higher WTP to the larger expected quality of life improvement, is not supported. We find WTP-Q values that conform reasonably well to previous studies in Sweden.

## Introduction

Total expenditure on health as a percentage of the gross domestic product in Sweden was about 11% in 2015, which was higher than the OECD average of 8.9% [1]. Although the share of health care expenditures in the GDP has increased in recent decades, prioritization between medical treatments and public health interventions has perhaps never been examined more carefully. Economic evaluations are an important input to decision-making and priority-setting in this sector, and measuring preferences for health improvements is one component in the interpretation of the results from health economic evaluations. The most commonly used tool for economic evaluation in health is cost-effectiveness analysis (CEA), where the incremental cost of an intervention is related to the incremental health effect (where the intervention is compared to the most relevant intervention alternative). The preferred/chosen metric of health benefits for a CEA is commonly quality-adjusted life years (QALYs).

Considering that costs and health benefits are measured in different units, the result can never in itself indicate whether an intervention improves welfare and/or population health. Two decision rules have been suggested when using CEA with QALYs (or similar) as an outcome measure [2]: choose interventions in ascending order of cost per QALY until the budget is exhausted or select interventions with a cost per QALY less than or equal to a specified threshold value (*V*). The second decision rule (“threshold approach”) is usually what policy makers rely on given that decisions are typically made sequentially in time. The decision rule can be written as: ${\text{if}}\,\,\Delta {\text{Cost}}/\Delta {\text{QALYs}} < V \to$ the intervention is cost-effective, i.e., if the incremental cost-effectiveness ratio (ICER) is below the threshold value.^1^

In policy contexts where the analyst only cares about heath care sector-related costs and benefits and assuming a fixed budget, the estimate of *V* should be based on the value (cost per QALY) of displaced services from implementing cost-increasing interventions. If the analyst considers a broader societal perspective and/or assumes a non-fixed budget, the estimate of *V* should be based on (or adjusted for) the consumption value of a QALY.

Attempts to assign a monetary metric to the consumption value one of a QALY can be conducted by eliciting the willingness to pay (WTP) for a QALY, henceforth referred to as WTP-Q (e.g., [3–9]). It is well known that the underlying theoretical assumptions to directly translate QALYs into monetary units, and treat QALYs as a utility metric, are restrictive [10–12].^2^ It has also been shown that under a range of admissible utility functions the WTP-Q will vary with the type of QALY gains [10, 13].^3^ The empirical evidence also provides evidence that the WTP-Q varies with the type of QALY gain. In a review of 24 previously published WTP-Q studies, it is shown that larger QALY changes give lower WTP-Q estimates and therefore that WTP is not proportional to the QALY change [14]. Several studies that have examined the sensitivity of scope, i.e., how willingness to pay changes with the amount of QALYs gained, have also found the same disproportionality [3, 7, 8, 15].

However, although the theoretical possibilities to estimate one unique WTP per QALY are insurmountable, there may still be valuable information to extract from studies on individual preferences for health improvements by the range of WTP-Q estimates in a population [13, 15].

In policy/jurisdictional contexts that evaluate cost-effectiveness of new interventions and medical technologies with respect to the consumption value of a QALY, it is rare to rely on one constant WTP-Q, but rather to have an interval of what possibly constitutes the relevant value of a QALY (e.g., [16]). Empirical estimates are thus important to provide insights for the potential bounds of such an interval of WTP-Q estimates.

This study adds to the previous literature in building knowledge about the estimate of WTP-Q, the variables influencing its size, and testing whether WTP is sensitive to changes in health as well as the level of uncertainty regarding the health improvement. WTP is estimated based on survey responses to a web-based contingent valuation survey. We test the sensitivity of the WTP to the magnitude of the health change as well as to the probability for health improvement and all valuation scenarios are framed in a decision context with uncertainty regarding the outcomes. This is standard in the literature on WTP for mortality risk reductions, for example, but most papers in the WTP-Q literature have used scenarios with choice under certainty (although with some recent exceptions [17]).

Specifically, we address the following two research questions: (1) if WTP increases with the amount of health difference and probability for improvement and (2) if WTP is approximately proportional to the magnitude of health difference and probability for improvement. We describe the methods and data in Sect. 2, where we also outline our specific hypotheses. Results are presented in Sect. 3, and the paper concludes with a discussion in Sect. 4.

## Methods and data

### Survey structure

We use data from an internet panel survey conducted in the spring of 2014. The full contingent valuation survey consisted of five sections as well as an introductory note to respondents. The five sections contain the following: (1) respondents' self-reported health status using a visual analog scale (VAS) between 0 and 100 as well as their views on the subjective health status of a number of different described health states,^4^ (2) a description of two health states (named A and B) where the respondent was asked about their individual WTP to move from the worst to the better health state, (3) a scenario where respondents were asked to act as social decision-makers “voting” yes or no to introducing/reimbursing a new pharmaceutical that would increase life expectancy at old age for terminally ill patients, (4) questions on respondents’ attitudes to different prioritization “rules,” e.g., if they agreed to different normative statements about how resources should be allocated, and (5) socioeconomics and demographics. Sections 3 and 4 are not analyzed in this article.

The survey consisted of 21 questions in total and was approved by the regional ethical vetting board. We tested the survey in small focus groups and subsequently in a pilot survey with approximately 200 respondents. As a result of the pilot survey, we modified the cost levels upwards and clarified the text further for specific sections (mainly by using extra bold type text to stress the importance of certain keywords).

### Scenario design

In this article we focus on the individual WTP for an improved health state (Sect. 2 of the survey). A general description of the WTP scenarios took the following form (the valuation scenario and WTP question are also presented in the Appendix). First, we asked the respondents to consider a possible treatment that is able to improve a specific health state. The treatment does not cause any pain, has no side effects, and is not subsidized by the society. We reminded the respondents about their opportunity cost, i.e., if they would choose to pay for the treatment they would have less money for food, travel, entertainment, clothes, etc. (“cheap-talk” script to reduce the risk of hypothetical bias). It was also assumed that the national insurance compensates for all possible health care costs and loss of income due to sickness in order for the responses not to be biased by perceived income changes (we want to estimate the “pure” value of health).

Second, the respondents were asked to assume that their health state today was equal to a specific EQ-5D state (health state A). Then, they were told that there is a 1% probability of health improvement by natural causes that would result in a better EQ-5D state (health state B). If they would choose to pay for the treatment, the probability of attaining the better health state would increase to 5%.^5^ Third, the respondents were asked if they were willing to pay SEK 20/200/500/1500/3000 per month^6^ over the next year for this treatment (yes/no).^7^ Finally, we included a certainty calibration question about how confident the respondents were about their response to the WTP question.^8^

The health states were chosen and paired to represent an expected good spread of QALY differences according to the UK EuroQoL tariffs [19]. We establish a ‘small,’ ‘medium,’ and ‘large’ health difference (Table 1). All have the same final health state, but different initial states.^9^ Since we did not have any Swedish EuroQoL scoring function at the time, we assumed that the UK values would represent the best approximation. In 2014, Burström et al. [20] published a Swedish value set for EQ-5D health states, and we compare the results in this respect as well. Further, from the results in Sect. 1 in the survey, we have the respondents’ self-assessed EQ-5D VAS tariffs for health states A and B, which we also analyse.

**Table 1:** The choice scenarios (UK TTO scores)

Choice scenario	Health state A		Health state B		Health diff	Probability diff (%)	QoL diff
	State	Score	State	Score			
Small	12,121	0.692	11,121	0.796	0.104	4	0.00416
Medium	22,222	0.516	11,121	0.796	0.280	4	0.0112
Large	12,223	0.151	11,121	0.796	0.645	4	0.0258
Small_scope	12,121	0.692	11,121	0.796	0.104	40	0.0416
Large_scope	12,223	0.151	11,121	0.796	0.645	40	0.258

The three health differences are paired such that five alternatives are established (Table 1). Three scenarios represent a value set where the probability for a better health state is increased by 4% points. Two scenarios represent a set to enable a distinct scope test regarding the uncertainty level, since the differences in health are the same as for scenarios ‘small’ and ‘large,’ but the probability for improvement is increased to 40%. The expected quality of life (QoL) difference is calculated as health difference multiplied by the probability difference. Each scenario was randomly presented to the respondents and each respondent only answered one valuation question.

The scenarios represent different baseline levels and magnitude differences between QALY scores. If we assume that “a QALY is a QALY is a QALY,” the null hypothesis would be that estimated WTP values are sensitive to both the health difference and the probability for improvement. Formally, we would expect that the following propositions hold [21]:

#### **Proposition 1**

*Willingness to pay increases with the amount of health difference and probability for improvement* (*weak scope sensitivity*).

#### **Proposition 2**

*For small changes, willingness to pay is approximately proportional to the magnitude of health difference and probability for improvement* (*strong scope sensitivity*).

If we assume that both propositions hold, we would expect WTP to be approximately proportional to the quality of life difference. Practically, this would imply that our hypotheses are:

1. WTP(’small’) < WTP (‘medium’) < WTP (‘large’) < WTP (‘small scope’) < WTP (‘large scope’).
2. 10 × WTP (‘small’) = WTP (‘small scope’).
3. 10 × WTP (‘large’) = WTP (‘large scope’).

Hypothesis (1) is based on proposition 1 of weak scope sensitivity, i.e., we expect WTP for a ‘small’ quality of life difference to be larger than a ‘medium’ quality of life difference, etc. (get more, pay more). Hypotheses (2) and (3) are based on the proposition of strong scope sensitivity, i.e., we expect WTP for a ‘small’/‘large’ quality of life difference to be ten times smaller than their scope alternatives. We expect WTP to be proportionally sensitive to the quality of life differences among the first three scenarios as well, i.e., ~3× WTP (‘small’) = WTP (‘medium’).^10^

### Data

Respondents were 1400 members of a web panel consisting of Swedish citizens older than 17 years. They were randomly recruited to the panel by phone and had to be internet users. The on-line survey was conducted in the spring of 2014 and was carried out by the Scandinfo company. The respondents were assigned at random to one of the scenarios described in Table 1.^11^

In Table 2, we show the summary statistics for the full sample as well as for the five individual scenarios. The self-reported health is based on the individual responses to the EQ-5D descriptive system. There are no statistically significant differences across the scenarios for any of the observable variables. Compared to national statistics, the sample has a higher share of individuals with university education of 3 years or more (33% compared to 20% in the population), a higher disposable monthly income (approximately SEK 32,000 compared to SEK 24,000), and a lower share of unemployed individuals (4.5% compared to 5.7%) [22–24]. The share of females and mean age correspond to the national sample.

**Table 2:** Descriptive statistics, mean (standard deviation)

Variable	Full sample	Small	Medium	Large	Small scope	Large scope
Number of respondents	848	168	180	168	178	176
Female	0.50 (0.50)	0.49 (0.50)	0.55 (0.50)	0.48 (0.50)	0.51 (0.50)	0.49 (0.50)
Age	44.3 (17.0)	43.9 (17.3)	42.7 (16.7)	47.3 (17.5)	43.7 (15.5)	45.1 (17.7)
University education	0.34 (0.47)	0.35 (0.48)	0.36 (0.48)	0.34 (0.48)	0.34 (0.47)	0.35 (0.48)
Unemployed	0.050 (0.22)	0.036 (0.19)	0.061 (0.24)	0.048 (0.21)	0.045 (0.21)	0.057 (0.23)
Income	32,087 (17,084)	33,035 (17,585)	32,333 (16,760)	31,071 (16,911)	33,413 (17,643)	34,134 (17,006)
Subjective health (EQ-5D)	0.85 (0.19)	0.84 (0.21)	0.86 (0.20)	0.85 (0.18)	0.84 (0.19)	0.85 (0.22)

We exclude some respondents based on the following reasons: respondents indicating an implausibly high number of children in the household (*n* = 36) and young respondents with an implausible high income or education level (*n* = 22). In Sect. 3.2, we also exclude respondents based on other inconsistencies as part of a sensitivity analysis.

## Results

### Proportions, WTP-Q, WTP, and determinants of WTP

The proportions of yes responses (Table 3) are decreasing for all samples and individual scenarios as the cost rises, although not monotonically for all. Notably, the scope scenarios still have a high proportion of yes responders (above 40%) at the highest bid level. We may also cross-compare the proportions between scope samples and their equivalent. Using a 95% confidence interval, there is no significant difference in proportions.

**Table 3:** Proportions of yes responses (in percent) at different bid levels

Bid level (SEK)	Full sample (incl. scope)	Full sample (excl. scope)	Small	Medium	Large	Small scope	Large scope
240	87 (*n* = 163)	87 (*n* = 90)	91 (*n* = 32)	88 (*n* = 32)	81 (*n* = 26)	82 (*n* = 28)	91 (*n* = 45)
2400	73 (*n* = 165)	69 (*n* = 106)	60 (*n* = 35)	65 (*n* = 31)	80 (*n* = 40)	75 (*n* = 24)	86 (*n* = 35)
6000	63 (*n* = 187)	57 (*n* = 113)	61 (*n* = 36)	52 (*n* = 42)	57 (*n* = 35)	72 (*n* = 40)	71 (*n* = 34)
18,000	39 (*n* = 182)	30 (*n* = 117)	29 (*n* = 35)	28 (*n* = 43)	33 (*n* = 39)	56 (*n* = 27)	56 (*n* = 39)
36,000	31 (*n* = 150)	24 (*n* = 90)	17 (*n* = 30)	34 (*n* = 32)	21 (*n* = 28)	42 (*n* = 26)	41 (*n* = 34)

*n* number of respondents

We estimate WTP-Q for each choice scenario by using a no-constant binomial logit model (Eq. 1), where the dependent variable was set to 1 if the respondents answered ‘yes’ to the WTP question and 0 if the answer was ‘no’ [13].^12^ The cost variable is multiplied by 12 to account for WTP on an annual basis. The value difference between health states A and B is multiplied by the risk reduction (4 or 40%) to calculate the effective quality of life differences (QoL_diff).

1 $$ y = \beta_{1}^{*} {\text{bid}} + \beta_{2}^{*} {\text{QoL}}\_{\text{dif}}f $$

and

2 $$ {\text{WTP}}/{\text{QALY}} = - \frac{{\beta_{2}^{*} }}{{\beta_{1}^{*} }} $$

Full sample estimates indicate a WTP-Q of SEK 167,947–373,979 including the scope sample (€17,100–$38,100) and SEK 749,189–1,153,523 excluding the scope sample (€76,400–€117,700), depending on the tariff used (Table 4). Scenario-specific estimates of WTP-Q range from SEK 104,091 to SEK 10,716,153 (€10,600–€1,093,500), clearly implying that the hypothesis of a constant WTP-Q value is not met. We can see that the WTP-Q estimate for the scenario ‘small’ is significantly higher than the rest of the scenarios and the estimate for the scenario ‘large scope’ is significantly lower, and the estimate for ‘small’ is significantly higher than for ‘small scope’ (UK and Swedish tariffs). For the Swedish tariffs, WTP-Q for the scenario ‘small scope’ is significantly higher than WTP-Q for the scenario ‘large.’ WTP-Q for the scenario ‘large scope’ is significantly lower than WTP-Q for the scenario ‘large’ for all tariffs. Estimates for the other VAS scenarios are not significantly separable, but the confidence intervals are very wide.

**Table 4:** Estimated WTP per QALY (SEK), logistic model

	Full sample (incl. scope)	Full sample (excl. scope)	Small	Medium	Large	Small scope	Large scope
UK EQ-5D tariffs							
WTP-Q	167,947	749,189	2,885,118	1,202,915	545,570	649,810	104,091
95% CI	122,642–213,252	546,247–952,132	1,934,743–3,835,493	702,751–1,703,079	377,870–713,270	386,779–912,841	76,443–131,739
Swedish EQ-5D tariffs							
WTP-Q	282,411	1,153,523	10,716,153	1,347,365	926,033	2,413,580	176,681
95% CI	202,052–362,770	841,588–1,465,457	7,186,188–14,246,118	787,081–1,907,449	641,384–1,210,683	1,436,609–3,390,552	129,752–223,610
Self-assessed EQ-5D VAS tariffs							
WTP-Q	373,979	1,037,392	1,020,158	1,079,550	974,710	762,511	285,603
95% CI	264,466–483,493	697,309–1,377,475	17,074–2,023,243	457,226–1,701,874	553,865–1,395,556	−7413 to 1,532436	152,096–419,110

Compared to the propositions (Sect. 2.2) estimated WTP-Q values are dependent on the quality of life differences, both the health difference itself and also the probability for improvement. If we present the isolated estimated WTP values for each scenario (QoL_diff*WTP-Q), we can see that the result implies that estimated WTP is lower for scenarios ‘small’ and ‘large’ than for their scope equivalents (Table 5). None of the other scenarios show significantly different WTP estimates between each other. We also calculate mean WTP by using a non-parametric method (Spearman-Karber) and achieved the same results.

**Table 5:** Estimated WTP (in SEK) for each choice scenario, logistic model

	Small	Medium	Large	Small scope	Large scope
Mean WTP	12,002	13,473	14,076	27,032*	26,855**
95% CI	8049–15,956	7871–19,074	9749–18,402	16,090–37,974	19,722–33,988
*N*	168	180	168	145	187

*N* number of respondents

*Significantly higher than scenario ‘small’

**Significantly higher than scenario ‘small,’ ‘medium,’ and ‘large’

Depending on the values respondents assess to different health states, we expect WTP to differ proportionately (proposition 2). Table 6 shows the quality of life differences and expected WTP differences for the UK tariffs, the Swedish tariffs, and the self-assessed VAS tariffs. We can see that the quality of life differences vary and that the hypotheses of proportional scope sensitivity are somewhat altered depending on which tariff is used.

**Table 6:** Quality of life differences and expected WTP differences

Choice scenario (S)	QoL_diff (UK)	Expected quota (UK)*	QoL_diff (SWE)	Expected quota (SWE)^a^	QoL_diff (self-assessed VAS)	Expected quota (VAS)^a^
Small	0.00416	1	0.00112	1	0.00416	1
Medium	0.0112	3	0.0100	9	0.0106	3
Large	0.0258	6	0.0152	14	0.0105	3
Small_scope	0.0416	10	0.0112	10	0.0426	10
Large_scope	0.258	62	0.152	136	0.0944	23

^a^Compared to the QoL difference for scenario ‘small’

Table 7 presents the estimated WTP (probability of a yes answer to the WTP question) by the samples and socio-demographic variables of interest in a logit regression. In general, we can see that neither gender nor age has a significant effect on WTP. Being unemployed, the income level, and having a university education have significance for some scenarios. As expected, we see a negative effect on the proportion of yes responses as the bid level increases (−11 to 23% percentage points per SEK 10,000).

**Table 7:** Estimated WTP (probability of a yes answer), marginal effects (logit model)

Variables	Full sample (incl. scope)	Full sample (excl. scope)	Small	Medium	Large	Small scope	Large scope
Gender	−0.038 (0.307)	−0.047 (0.339)	−0.12 (0.221)	0.015 (0.856)	−0.057 (0.525)	−0.075 (0.391)	0.037 (0.608)
Age (10 years)	−0.016 (0.144)	−0.013 (0.353)	0.024 (0.393)	−0.049* (0.053)	−0.013 (0.610)	−0.023 (0.403)	−0.021 (0.311)
Income (SEK 100,000)	0.23** (0.044)	0.15 (0.303)	−0.036 (0.894)	0.44* (0.092)	−0.023 (0.931)	0.24 (0.354)	0.33 (0.130)
Bid (SEK 10,000)	−0.16*** (0.000)	−0.19*** (0.000)	−0.23*** (0.000)	−0.15*** (0.000)	−0.21*** (0.000)	−0.11*** (0.000)	−0.13*** (0.000)
Unemployed	−0.0034 (0.970)	−0.051 (0.622)	−0.26 (0.121)	−0.019 (0.918)	0.076 (0.618)	−0.43* (0.079)	0.20** (0.010)
University education	0.068* (0.091)	0.12** (0.022)	−0.0079 (0.932)	0.12 (0.198)	0.26*** (0.007)	−0.070 (0.459)	0.094 (0.193)
Subjective health (EQ-5D)	0.10 (0.305)	−0.030 (0.829)	−0.33 (0.254)	0.18 (0.512)	−0.034 (0.889)	0.52 (0.101)	0.18 (0.336)
Number of respondents	847	515	168	180	167	145	187

*, ** and *** denote statistical significance at the 10, 5 and 1% level, respectively. Based on robust standard errors, *p* values in parentheses

### Sensitivity analysis using certainty calibration and excluding inconsistent respondents

We have tested the results in the previous sections in two different sensitivity analyses to account for uncertainties in individual responses: (1) by using certainty calibration and (2) excluding inconsistent respondents. Hypothetical bias is found to be a serious problem of CV data, and incorporating respondent uncertainty can potentially improve the predictive power (e.g., [25, 26]). We have used a version of the certainty approach that follows up on the WTP question by letting the respondents assess the degree of uncertainty with three statements: ‘definitively sure,’ ‘probably sure,’ and ‘uncertain.’ Only the ‘definitely sure’ yes responses were treated as yes responses, while the ‘probably sure’ yes responses and the ‘uncertain’ yes responses were treated as no responses. No treatment was carried out with the no responses.

Of the 499 yes responses to the WTP question, 249 (i.e., 50%) stated they were ‘definitely sure.’ We can see the same decreasing proportions of yes responses after certainty calibration for all samples as the cost rises, as we saw in Table 3, although the proportions are naturally lower (since we convert yes responses to no responses). The estimated WTP-Q values are much lower and are not significantly different for any sample, nor are there any significant differences between any WTP values, i.e., neither weak nor strong scope sensitivity.

Based on our subjective assessment of responses, we believe that some respondents gave answers of low quality. They may not have understood the survey, or they may have considered it not to be worthwhile to leave a thoughtful response. The inconsistent respondents were defined as someone that: (1) rated the subjective health status of being dead higher than having ‘perfect health’ (*n* = 65), (2) rated the subjective health status of being dead higher than 50 on a VAS (*n* = 113), or (3) rated the better health state lower than the worse health state on a VAS (*n* = 73). Some overlapping existed, but 208 individual respondents were deleted.

The proportions of yes responses are decreasing for most bid levels. WTP-Q values for the adjusted sample are generally higher for all three tariffs than in Table 4, which indicates that the deleted respondents have lower WTP. The samples show significantly different WTP-Q values, especially between ‘large’ and ‘large scope’ (all tariffs). Estimated WTP is significantly higher for the scenario ‘large scope’ than for scenarios ‘small,’ ‘medium,’ and ‘large.’ The quotas are approximately two, implying weak but not strong scope sensitivity.

The last model in the sensitivity analysis combines certainty calibration with the exclusion of inconsistent respondents. The WTP-Q values are, in general, slightly higher for UK and Swedish tariffs, but lower for the self-assessed VAS tariffs. None of the scenario-specific WTP-Q or estimated WTP values were significantly different from another.

## Discussion

Our article addressed one specific general research question: is WTP sensitive to the size of the health differences and the probability for improvement? We also examined what socio-demographic factors are related to the variations in WTP and what the willingness to pay per quality-adjusted life year (WTP-Q) in Sweden is. To answer these questions, we used data from an internet panel contingent valuation survey conducted in the spring of 2014. The results are based on 1400 respondents, and they were randomly blocked into different scenarios, where the health differences as well as the probability for improvement were varied.

The survey results show that the estimated WTP-Q ranges between SEK 170,000 (UK tariffs) to SEK 370,000 (self-assessed VAS tariffs). Swedish EQ-5D tariffs result in intermediate WTP-Q values of SEK 280,000. Previous WTP-Q values in Sweden have been estimated to range between SEK 400,000–655,000 [27, 28]. One study, with a low response rate of ~12%, estimates WTP-Q to be in the wide interval SEK 100,000–1,900,000 [29]. A threshold value of SEK 500,000 has been suggested by government authorities [30]. In an international review of 24 WTP-Q studies, mean estimates amount to approximately SEK 700,000 [14].

However, our analyses looking at the sub-samples and addressing the questions of sensitivity to scope showed that the empirical results do not conform to necessary assumptions of the QALY concept. Our results showed that the prediction of expected utility theory and, more importantly, a standard assumption saying that the more QALYs the better, i.e., that WTP increases with the amount of quality of life improvements (weak scope sensitivity) or ‘more is better,’ can only be partially supported for the largest improvements (ratios above 10, and not always even then). We see no support for approximate proportionality (strong scope sensitivity), implying that we cannot empirically establish a constant WTP-Q value. The hypothesis set out was that WTP-Q values should be the same, while WTP values should differ depending on the health difference and probability for improvement, i.e., WTP varies with the QALY difference. The result was basically the reverse: WTP for the different QALY changes did not vary in any substantial way, and thus WTP-Q estimates vary substantially. Considering that WTP-Q = WTP/QALY difference and that WTP does not vary with the QALY difference, this implies that larger QALY changes give lower WTP-Q estimates.

The result points to an inadequate sensitivity in WTP to scope (scope bias), which is often reported in contingent valuation studies and stated preference approaches in general (e.g., [31, 32]). Several studies regarding WTP per QALY have found evidence of scope bias [3, 4, 7, 8, 15], and a recent meta-analysis estimate a 64% decrease in WTP-Q per unit larger QALY change [14].

The low responsiveness to the changes in quality of life (QoL) differences, to changes in both health and probability for improvement, is problematic given the policy aim of pursing to find the populations’ consumption value of a QALY. There is a risk that WTP-Q values rest on the specification of the health differences and the probability of improvement, which is often set by a researcher for a specific design. We have specified large and small variations in QoL between the scenarios, and the empirical results show that WTP-Q is far from constant. In our sensitivity analysis, we have used certainty calibration and excluded inconsistent respondents. The results were that WTP-Q values are not statistically significantly different over the scenarios, but neither are the estimated WTP values. The confidence intervals are very wide, which explains this result.

We acknowledge that sensitivity to scope is one test of the validity of CV, but not the only one. We can see that the proportion of yes responses decreases with the cost level and there are some variations in WTP comparing the lowest and highest QoL differences (weak scope sensitivity). If the true QALY model is in fact non-linear, we would not expect the assumptions about expected utility to reflect respondents’ preferences [33–35]. We also acknowledge that the conventional scope test, i.e., comparing mean values, can hide important relationships and lead to false positives and false negatives [36].

The comparison among the three separate tariffs is another interesting aspect of this study. Most previous studies have used one single tariff or individual self-assessed QoL estimates. We have no reliable information on which tariff most accurately reflects the respondents’ preferences and, as we saw earlier (Table 6), the expected ratios between different scenarios are very dependent on this. As the estimated WTP values in scenarios ‘small,’ ‘medium,’ and ‘large’ are not significantly different, this would lead us to consider that self-assessed VAS tariffs are most likely to reflect the invariance in WTP values. In this case, the ratios are between 0.7 and 3, while for the Swedish tariffs the ratios are 1–14. Hence, it is easier to justify that an expected WTP difference of 3 does not arise than an expected difference of 14. In sum, the result that the chosen tariff substantially affects the WTP-Q is not particularly convincing for the attempts to elicit an empirically reliable WTP-Q.

Our examination of determinants of WTP (Table 7) results in the conclusion that the only variable that is significant thorough all of the scenarios is the cost. There are some significant associations for specific scenarios, but the variable differs. Income and university education had a positive significant effect for the full sample, but not for specific scenarios.

Finally, a number of important study limitations should be mentioned. Compared to the Swedish population, the sample had a higher income and share of university level education, which implies that the generalizability can be questioned. However, given that the primary aim was not to elicit policy estimates of WTP-Q, the fact that the different subsamples that we used for comparison did not significantly differ from each other is assuring. Further, it is well known that it is difficult to intuitively communicate small changes in probabilities. Failure to understand our survey scenario could of course be one argument as to why we fail to find adequate sensitivity to scope.

To conclude, we found that our expectation of sensitivity to scope, or higher WTP with the larger expected QoL improvement, was not supported. We have also documented that the willingness to pay per QALY was substantially affected by the chosen tariff to estimate QoL. Even though a single WTP-Q may not be theoretically or empirically attainable, we believe that a threshold based on state-of-the-art research from several data sources will help to improve efficiency in society. However, this article, as well as the broader literature on this topic, struggles to provide estimates that pass the contingent valuation (or stated preferences) validity tests, including the near proportionality of willingness to pay.

## Appendix

### The EuroQoL descriptive system (EQ-5D)

**Table:**

Dimension	Level	Description
Mobility	1	I have no problems in walking about
	2	I have some problems in walking about
	3	I am confined to bed
Self-care	1	I have no problems with self-care
	2	I have some problems washing or dressing myself
	3	I am unable to wash or dress myself
Usual activities (e.g., work, study, homework, family or leisure activities)	1	I have no problems with performing my usual activities
	2	I have some problems with performing my usual activities
	3	I am unable to perform my usual activities
Pain/discomfort	1	I have no pain or discomfort
	2	I have moderate pain or discomfort
	3	I have extreme pain or discomfort
Anxiety/depression	1	I am not anxious or depressed
	2	I am moderately anxious or depressed
	3	I am extremely anxious or depressed

### The valuation scenario and WTP question (translated from Swedish)

Part 2: How do you value different health states?

Now we want to know how much you think it might be worth it to pay for a treatment that can improve a supposed state of health. Imagine that the treatment does not hurt, does not have any side effects, and is not paid for by the community.

Remember, if you think that the treatment is worth the money it means that you and your family get less money for other things, such as food, travel, entertainment, and clothing. We also assume that the social security system compensates you for any medical expenses and loss of income in case of illness.

<NEW SCREEN>

Consider the following scenario:

Your health state today can be described as the left below (health state A).

There is a chance of 1 in 100 that your health is improved by natural causes during the coming year. Your health would then be described as the right below (health state B). If you imagine that you are part of a group of 100 people, this means that one of you will get an improved state of health in the coming year. But beforehand, no one knows who is going to get better.

There exists a treatment that increases the chance of achieving better health, does not hurt, and does not have any side effects.

If you have the treatment, your chance to a better level of health is instead 5 in 100.

Note that it is your personal chance that is affected, not the chance for any of the others in the group. They choose for themselves if they want to have the treatment.

**Table:**

Health state A	Health state B
I have no problems in walking about [1] I have some problems washing or dressing myself [2] I have no problems with performing my usual activities [1] I have moderate pain or discomfort [2] I am not anxious or depressed [1]	I have no problems in walking about [1] I have no problems with self-care [1] I have no problems with performing my usual activities [1] I have moderate pain or discomfort [2] I am not anxious or depressed [1]

**Question 7** Would you be willing to pay [SEK 20/200/500/1,500/3,000 per month] over the next year for this treatment that increases the chance of a better level of health from 1 in 100 to 5 in 100?

□ Yes □ No

## Footnotes

1 Or as is also common in the net monetary benefit perspective, if $\Delta {\text{QALYs}} \times V - \Delta {\text{Cost}} > 0 \to {\text{cost - effective}}.$

2 The following conditions have to be met for QALYs to represent a valid utility function: (1) mutual utility independence, (2) constant proportional trade off of longevity for health, (3) risk neutrality over life span, and (4) additive independence across periods [11].

3 A constant WTP-Q value would make it theoretically valid to estimate the monetary gain of a health improvement by multiplying the gain in QALYs with a WTP-based value of a QALY.

4 The subjective health states that the respondents were asked to rate with the VAS were: the status today, being dead, having ‘perfect health,’ and two predetermined health states (A and B).

5 The uncertainty in health improvement by natural causes and by treatment aims to better reflect reality.

6 €1 = SEK 9.8 (May 2017).

7 The dichotomous choice format (“yes or no purchase decision”) was chosen since it has been shown to be the response format in CV studies (compared to, e.g., open-ended or payment card elicitation techniques) that is potentially incentive-compatible [18].

8 The certainty follow-up alternatives were: ‘definitively sure,’ ‘probably sure,’ and ‘not sure.’

9 We also included one sub-sample with a lower final health level. This scenario does show that the WTP estimates are stable independent of where on the QALY scale the health differences are positioned. This sample is not further included in the analysis.

10 Based on the QoL differences (0.0112/0.00416 ~ 3).

11 One-sixth of the full sample was assigned into the lower final health level scenario, which is not included in this analysis.

12 For the individual scenario, the QoL_diff is constant, i.e., for the scenario ‘small’ the QoL_diff is equal to 0.00416 (UK EQ-5D tariffs).
